# The rs9953490 polymorphism of DAL-1 gene is associated with gastric cancer risk in the Han population in Northeast China

**DOI:** 10.1186/s12876-021-01929-9

**Published:** 2021-09-27

**Authors:** Hui Wang, Yuling Jiang, Lina Yu, Lidan Xu, Rongwei Guan, Mengdi Cai, Kexian Dong, Xiao Liang, Jing Bai, Jingcui Yu

**Affiliations:** 1 Scientific Research Centre, The Second Affiliated Hospital of Harbin Medical University, Harbin, 150081 China; 2 Department of Blood Transfusion, The Second Affiliated Hospital of Harbin Medical University, Harbin, 150081 China; 3 The Clinical Laboratory, The First Affiliated Hospital of Harbin Medical University, Harbin, 150001 China; 4 Key Laboratory of Preservation of Human Genetic Resources and Disease Control in China (Harbin Medical University), Ministry of Education, Harbin, 150081 China

**Keywords:** Gastric cancer, DAL-1, Single nucleotide polymorphism, Susceptibility

## Abstract

**Background:**

DAL-1 gene was reported to inhibit proliferation, migration, invasion, and epithelial to mesenchymal transition (EMT) of gastric cancer (GC) cells in our previous study. The association between the genomic variants in DAL-1 gene with risk of GC is still unclear.

**Methods:**

In this study, 505 GC cases and 544 healthy controls (HCs) were collected to evaluate the association between six single nucleotide polymorphisms (SNPs) (rs7240736, rs73937194, rs3817466, rs8082898, rs73381527, rs9953490) of DAL-1 gene and GC risk in the Han population in Northeast China.

**Results:**

The TA + AA genotypes of rs9953490 were significantly associated with an increased risk in N3 compared with N0 subgroup (adjusted OR = 4.56, 95% CI = 1.49–13.98, *P* = 0.008), and also showed evident association with an increased risk in TNM stage III compared with stage I-II (adjusted OR = 2.33, 95% CI = 1.16–4.67, *P* = 0.017).

**Conclusion:**

The rs9953490 of DAL-1 gene may play an important role in the occurrence and development of GC in the Han population in Northeast China.

**Supplementary Information:**

The online version contains supplementary material available at 10.1186/s12876-021-01929-9.

## Background

Gastric cancer (GC) is the fifth most common neoplasia and the third leading cause of cancer-related death worldwide [1]. The histological heterogeneity classified GC into different subtypes as cardia carcinoma and noncardia carcinoma, which show distinct clinical, epidemiological and molecular features among [2], and seriously hinder the diagnosis and treatment [3]. Moreover, the incidence of GC is highly determined by multiple environmental factors as: microbial infections, the host genetic background (age, gender, lifestyle, dietary regime) [4, 5], alcohol, processed meat, and obesity [6]. Notably, effective diagnostic markers and targeted therapy against GC are still lacking. Currently, GC remains to be a serious fatal disease with poor prognosis throughout Asia, especially in China [7].

DAL-1 (differentially expressed in adenocarcinoma of the lung-1) gene is located on human chromosome 18p 11.3 and belongs to the 4.1 protein superfamily, which is isolated and detected in lung adenocarcinoma for the first time by *Tran *et al. [8]. The expression of DAL-1 is significantly reduced or lost in various tumors such as breast [9], hepatocellular [10], colon [11], and ovarian [12]. Several studies have identified that DAL-1 is significantly associated with cancer cell differentiation, lymph node metastasis, disease progression, and TNM stage [13, 14]. Our previous study first identified the loss of heterozygosity (LOH) at 18p11.3 region (DAL-1 loci) in 45 sporadic GCs, suggesting DAL-1 might be a candidate tumor suppressor gene [15]. Afterwards, we found promoter methylation-mediated down-regulation of DAL-1 in four GC cell lines and 94.6% (35 of 37) of surgically resected primary GCs. We also demonstrated that DAL-1 effectively inhibited the malignant transformation of GC cells [16], and was significantly associated with cancer progression and poor survival of GC patients [17].

Previous studies indicated that genomic variations may have great impact on the liability for GC [18]. Patients who carry the GG genotype of rs1049174 of NKG_2_D gene have a higher incidence of GC in Fujian Province of China [19]. Xu et al. [20] found that SNP rs4880 of SOD2 gene and SNP rs1695 of GSTP1 gene may increase cancer progression and tumor aggressiveness. Recently, we found that two SNPs (rs34700818 and rs61482741) of TOB1 gene were significant associated with increased GC progression [21]. Therefore, we investigated the association between six SNPs of DAL-1 gene and GC risk in a cohort of 505 GC patients and 544 healthy controls (HCs). Our data suggested that rs9953490 of DAL-1 might be an important marker of GC risk in the Han population of Northeast China.

## Material and methods

### Study population

505 unrelated Han Chinese primary GC patients who were recruited from Harbin Medical University Cancer Hospital, and 544 age- and sex-matched HCs who were recruited from the Second Affiliated Hospital of Harbin Medical University between January 2015 and June 2016, were included in the study. The ethics committee of the Second Affiliated Hospital of Harbin Medical University approved the trial protocol. Each participant was interviewed face-to-face by trained interviewers using a standardized questionnaire and signed a written informed consent. Peripheral blood was collected and stored at −80 °C for DNA extraction.

### SNP selection

The candidate SNPs were selected using the dbSNP database (http://www.ncbi.nlm.nih.gov/snp) and the 1000 Genomes database (https://www.ncbi.nlm.nih.gov/variation/tools/1000genomes/). These SNPs were selected based on the criterion of the minor allele frequency (MAF) greater than 0.05 in the Chinese Han population [21]. Ultimately, six SNPs (rs7240736, rs73937194, rs3817466, rs8082898, rs73381527 and rs9953490) of DAL-1 gene met the requirement and were included in our study in Table 1.

**Table 1:** Summarization of SNP information of DAL-1 gene

NO	Chr	SNP	Location	MAF (dbSNP)	MAF (1000 g-Genomes)
1	18	rs7240736	5'-flanking	0.39	0.49
2	18	rs73937194	5'-flanking	0.13	0.05
3	18	rs3817466	exon15	0.21	0.09
4	18	rs8082898	intron14	0.07	0.06
5	18	rs73381527	intron3	0.07	0.09
6	18	rs9953490	3'UTR	0.08	0.06

*Chr* chromosome, *MAF* minor allele frequency

### Genotyping

Genomic DNA was extracted from the blood samples using the Qiagen Blood DNA Mini Kit (Qiagen, Germany) according to the manufacturer's instructions. The improved Multiple Ligase Detection Reaction (iMLDR) method was used to acquire the genotypes of all the SNPs. The primer sequences of the six SNPs are shown in Table 2. Polymerase chain reactions (PCRs) were performed in a 20 μl reaction solution containing 1 µl of template DNA, 0.5 µl of each primer, 0.2 μl Taq enzyme (Qiagen, Germany) and 10 μl PCR reaction buffer (Takara, Japan). The original data were collected by ABI3730XL sequencer (Applied Biosystems, USA) and analyzed by GeneMapper 4.1 software (Applied Biosystems, USA). Genotyping data were confirmed with 10% randomly-selected samples which showed 100% concordance in repeated tests.

**Table 2:** The primer sequence for the six SNPs

Gene	SNPs	Primer sequence
DAL-1	rs7240736F	GGTCAGAGCCACTGTCCACTTG
DAL-1	rs7240736R	CAGCCTAGTTGTGGGCTGGAC
DAL-1	rs73381527F	CAGGCATTAGTCTTCATGCCATAAAAT
DAL-1	rs73381527R	TCTGTTGCTTTGGTTTATCATTTTTCA
DAL-1	rs8082898F	CTCAGGGAGCTGCAAGGAGAAG
DAL-1	rs8082898R	CCAGGTTATGAGCCGTCCAGAG
DAL-1	rs9953490F	ACATTCACCATGGGCTGTGATG
DAL-1	rs9953490R	AGAAACTGCTGGGCTTCCTGTG
DAL-1	rs3817466F	TAAACACAGAGCCACCCCACAA
DAL-1	rs3817466R	GACCTGTGCCAGAGCGTGTTTA
DAL-1	rs73937194F	GGTCAGAGCCACTGTCCACTTG
DAL-1	rs73937194R	GTCCAGCCCACAACTAGGCTG

*F* forward primer, *R* reverse primer

### Statistical analysis

Continuous variables with a normal distribution were described as mean ± SD and compared with the Student’s t-test [21]. Discrete variables we re described as frequency (percentage) and compared using the Chi-square (χ^2^) test [21]. Genotype frequencies for each polymorphism in the control group were tested by Hardy–Weinberg equilibrium using the χ^2^ test. Associations between the genotypic or allelic frequency and the risk of GC were estimated by odds ratios (ORs) and 95% confidence intervals (CIs). The age of the two groups at the stratified analysis was dichotomized according to the median age (58 years) of the control group [22]. Linkage disequilibrium (LD) and haplotype analyses were performed with Haploview 4.2 software (http://sourceforge.net/projects/haploview/). *P* values and ORs with 95% CIs were calculated using multiple regression analysis adjusted for age, gender, smoking status, pack-years, and drinking status. All statistical analyses were conducted using the SAS 9.3 software. All *P* values were two-sided, and *P* < 0.05 was considered statistically significant.

## Results

### Population characteristics

The data in Table 3 (at the end of the manuscript) showed the general information of all subjects of 505 GCs and 544 HCs. The mean age was 59.08 (59.08 ± 10.55 years) in GC group and 58.33 (58.33 ± 11.55 years) in the HCs. According to the 7th Edition of the American Joint Committee on Cancer (AJCC) [23], 87 cases (17.2%), 156 cases (30.9%), 128 cases (25.3%), 65 cases (12.9%) and 69 cases (13.7%) were classified as TNM I, II, III, IV and other stages, respectively. The number of subjects with a history of GC, no family history of cancer, and other cancers were 398 (78.8%), 42 (8.3%) and 65 (12.9%), respectively. There was no significant difference in age or gender between the two groups (*P* = 0.105 and *P* = 0.404), indicating no sample matching bias between groups. However, there was a significant difference in smoking status, pack-years and drinking status between the two groups (*P* < 0.0001).

**Table 3:** Clinical and demographic characteristics of cases and controls

Variables	Case, n (%)	Control, n (%)	*P*^a^
All subjects	505 (100.0)	544 (100.0)	
Age	23–92	25–87	0.105
Mean^b^	59.08 ± 10.55	58.33 ± 11.55	
≤ 50	107 (21.2)	129 (23.7)	
51–60	156 (30.9)	166 (30.5)	
61–70	168 (33.3)	170 (31.3)	
≥ 71	74 (14.6)	79 (14.5)	
Gender			0.404
Male	370 (73.3)	386 (71.0)	
Female	135 (26.7)	158 (29.0)	
Smoking status			< 0.0001*
Never	239 (47.3)	418 (76.8)	
Ever	266 (52.7)	126 (23.2)	
Drinking status			< 0.0001*
No	305 (60.4)	415 (76.3)	
Yes	200 (39.6)	129 (23.7)	
Pack-years			< 0.0001*
0	239 (47.4)	418 (76.8)	
≤ 25	77 (15.2)	27 (5.0)	
> 25	189 (37.4)	99 (18.2)	
Neoplasia location			
GCA	70 (13.9)	–	
NGCA	434 (85.9)	–	
ELSE	1 (0.2)	–	
Lauren’s classification			
Intestinal	203 (40.2)	–	
Diffuse	71 (14.1)	–	
Mixed	81 (16.0)	–	
Else	150 (29.7)	–	
TNM stageI			
I	87 (17.2)	–	
II	156 (30.9)	–	
III	128 (25.3)	–	
IV	65 (12.9)	–	
Else	69 (13.7)	–	
Family history of cancer			
None	398 (78.8)	–	
Gastric cancer	42 (8.3)	–	
Other cancer	65 (12.9)	–	

*GCA* gastric cardia adenocarcinoma, *NGCA* non-gastric-cardia adenocarcinoma

^*^indicate statistically significant data

^a^Two-sided χ^2^ test for distributions between cases and controls

^b^Data are mean ± SD

### Distribution of the genotypic and allelic frequencies of DAL-1 polymorphisms and their association with GC susceptibility

The genotypic frequencies of six SNPs except rs7240736 of the controls were all in accordance with Hardy–Weinberg equilibrium (*P* > 0.05). Hence the rs7240736 was eliminated in the next analysis. The genotypic distributions of the other five SNPs among the cases and controls and their associations with GC risk are summarized in Additional file 1: Table S1. All the allelic frequencies were not significantly different between the GCs and HCs. There was no evident association between the five SNPs with GC risk in the homozygotes, heterozygotes or two genetic models (dominant genetic model and recessive genetic model) after adjusting for age, gender, smoking status, pack-years, and drinking status (*P* > 0.05). The above comparisons were all not statistically significant by using multiple test correction.

### Haplotype analysis and GC risk

**Fig. 1:**
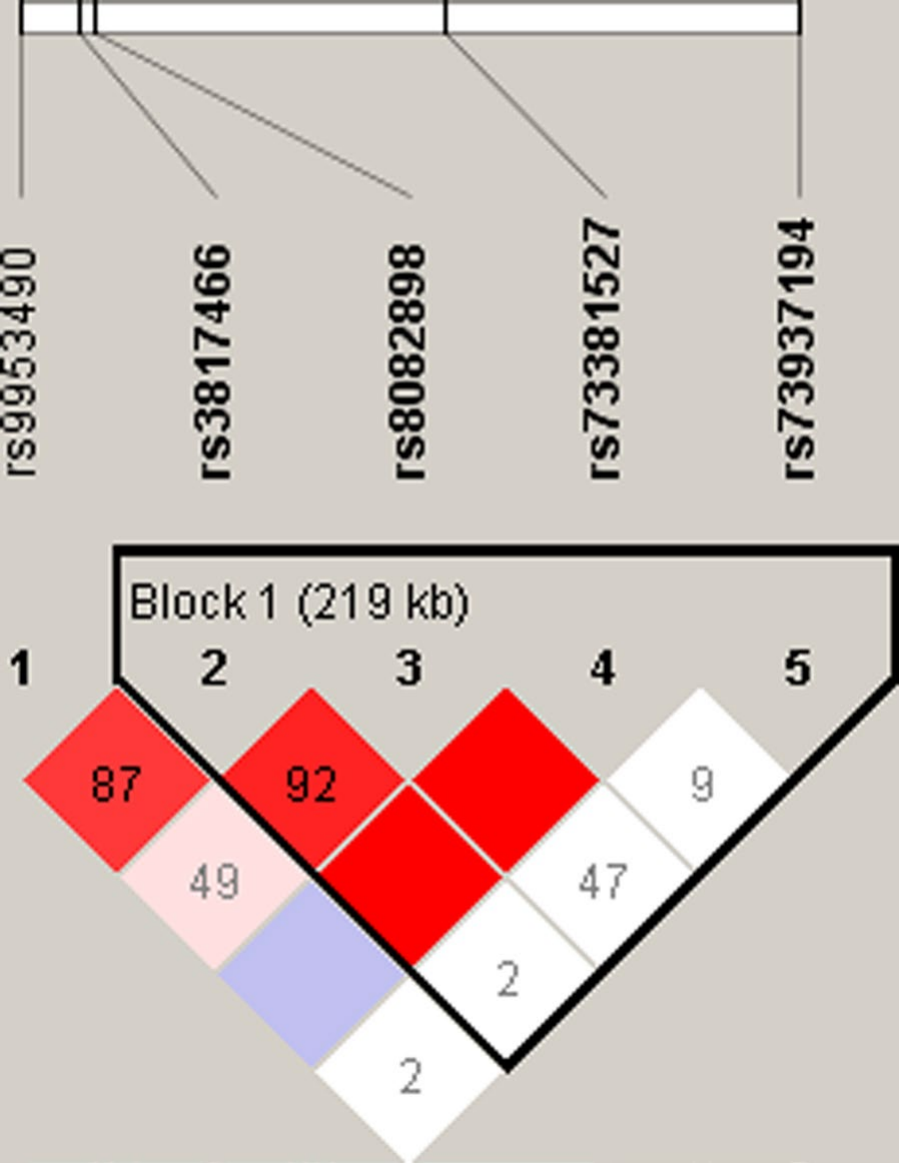
Linkage relationship and haplotype block in DAL-1 gene

No strong LD for the selected SNPs of the DAL-1 were identified by the Haploview software (Fig. 1). One LD block was found in the DAL-1 gene, which contained five haplotypes in the block. The associations between haplotype frequencies and GC risk were shown in Additional file 1: Table S2. The most common haplotypes within this block were determined as GTTC (0.777), ACTC (0.074), GTCC (0.068), followed by GTTG (0.044), and ATTC (0.020). No apparent association between each haplotype and GC risk within this block was observed.

### Stratified analysis between DAL-1 polymorphisms and GC risk

We conducted stratified analyses for all candidate SNPs according to age, gender, smoking status, pack-years, and drinking status, which were reported to have potential influences on GC occurence. As was shown in Table 4 (at the end of the manuscript), the TA + AA genotypes of rs9953490 were associated with a significantly higher GC risk in smoker than nonsmoker before adjustment by age, gender, smoking status, pack-years, and drinking status (TA + AA vs TT, OR = 2.33, 95% CI = 1.11–4.87, *P* = 0.025). However, this significant association was compromised after adjustment by age, gender, smoking status, pack-years, and drinking status (TA + AA vs TT, adjusted OR = 1.62, 95% CI = 0.61–4.27, *P* = 0.333). Meanwhile, for other SNPs, no significant association was found between genotypes and GC risk in the stratified analyses (Additional file 1: Tables S3–S7).

**Table 4:** Stratified analyses for the rs9953490 genotypes of DAL-1 gene in cases and controls

Variables	rs9953490 (cases / controls)		OR (95% CI)	*P*	Adjusted OR (95% CI)^a^	*P*^a^
	TA + AA (n)	TT (n)				
Age						
≤ 58	33/31	198/233	1	0.400	1.23 (0.70–2.14)	0.470
> 58	33/33	241/247		0.913	1.05 (0.59–1.86)	0.875
Gender						
Male	53/45	317/341	1	0.275	1.32 (0.83–2.10)	0.236
F emale	13/19	122/139		0.512	0.72 (0.31–1.69)	0.451
Smoking status						
Nonsmoker	25/55	214/365	1	0.319	0.85 (0.50–1.44)	0.537
Smoker	41/9	225/115	2.33(1.11–4.87)	0.025*	1.62 (0.61–4.27)	0.333
Pack-years						
0	25/48	214/370	1	0.688	0.85 (0.50–1.44)	0.537
≤ 25	9/4	68/23		0.673	1.40 (0.15–13.40)	0.768
> 25	32/12	157/87		0.281	2.31 (0.62–8.53)	0.210
Drinking status						
Nondrinker	42/50	263/365	1	0.494	1.19 (0.75–1.90)	0.466
Drinker	24/14	176/115		0.751	0.83 (0.37–1.86)	0.652

*CI* confidence interval, *OR* odd ratio

^*^indicate statistically significant data

^a^Adjusted for age, gender, smoking status, pack-years and drinking status

We further conducted stratified analyses in 274 patients with available clinicopathological information such as family history of cancer, tumor size, neoplasia location, depth of invasion, lymph metastasis, TNM stage, and Lauren’s classification. As shown in Table 5 (at the end of the manuscript), the TA + AA genotypes of rs9953490 were significantly associated with increased GC risk in N3 compared with N0 (TA + AA vs TT adjusted OR = 4.56, 95% CI = 1.49–13.98, *P* = 0.008), and with a obviously increased GC risk in TNM stage III compared with stage I-II (TA + AA vs TT adjusted OR = 2.33, 95% CI = 1.16–4.67, *P* = 0.017). No associations were found between other SNPs and the clinicopathological characteristics of GC (Additional file 1: Tables S8–S12). The above comparisons showed no statistical significance using multiple test correction analysis.

**Table 5:** Association between DAL-1 (rs9953490) genotypes and clinicopathologic features of GC

Variables	rs9953490		OR (95% CI)	*P*	Adjusted OR (95% CI)^a^	*P*^a^
	TA + AA(n)	TT (n)				
Tumor size(cm)						
< 5	21	140	1		1	
≥ 5	21	92		0.21	1.52 (0.77–2.98)	0.224
Neoplasia location						
Non-cardia	36	202	1		1	
Cardia	6	30		0.811	1.14 (0.43–2.99)	0.796
Invasion depth						
T1–T2	14	76	1		1	
T3-T4	28	156		0.942	0.94 (0.46–1.91)	0.863
Lymph metastasis						
N0	15	98	1		1	
N1	9	63		0.879	1.32 (0.51–3.41)	0.561
N2	10	54		0.666	1.49 (0.6–3.71)	0.388
N3	8	17	3.07(1.17–8.10)	0.023	4.56 (1.49–13.98)	0.008*
TNM stage						
I–II	23	167	1		1	
III	19	65	2.12(1.09–4.12)	0.026	2.33 (1.16–4.67)	0.017*
Lauren’s classification						
Intestinal	32	171	1		1	
Diffuse	10	61		0.735	0.87 (0.38–1.99)	0.738

*CI* confidence interval, *OR* odd ratio

^*^Indicates statistically significant data

^a^Adjusted for age, gender, smoking status, pack-years and drinking status

## Discussion

In the present study, we investigated the associations between six SNP polymorphisms of the DAL-1 gene and GC risk in the Han population in Northeast China. Stratification analyses based on smoking revealed that the TA + AA genotypes of rs9953490 were significantly associated with a significantly higher GC risk in smoker than nonsmoker. However, this association was abolished after adjustment by age, gender, smoking status, pack-years, and drinking status.

The reason might be as the following: First, although many studies have shown that smoking can increase the risk of GC [24, 25], the relationship between genomic polymorphisms and susceptibility to smoking-related GC has not yet been defined. A Meta-analysis about CYP1A1 polymorphisms with GC susceptibility pointed out that the m1 genotypes (CC + CT) decreased the susceptibility of GC among ever-smokers, but there wasn’t any association between the m2 genotypes (GG + AG) and GC risk among the smokers [26]. Second, the mechanism by which tobacco smoke facilitates cancer development is not completely elucidated. Moreover, most smoke-derived procarcinogens and their bioactivation are not organ-specific. When all tobacco-related cancers are considered together, smoking tends to increase the risk for developing cancers which are cancer-prone [24, 26, 27]. Third, the reason may also be due to the smaller sample size caused by subgrouping in stratified analysis, and the great discrepancy of sample number between subgroups. Last but not the least, GC is a heterogeneous disease involved with multiple etiological factors including age, gender, geography, lifestyle, dietary regime [4, 5, 28]. To confirm the results of this study, larger sample size will facilitate the statistic power so as to rule out the possibility of population stratification and “observation bias” [26] in future study.

Interesting, we found that the TA + AA genotypes of the rs9953490 were associated with the significant increased GC risk in N3 and TNM stage III, representing its possible correlation with lymph node metastases and poor prognosis of GC. We speculated that the TA + AA genotype, located in the 3’UTR region of the DAL-1 gene, might be able to regulate DAL-1 expression or its promoter methylation status, which may inhibit its original tumor suppressive function. In this case, the association of DAL-1 gene polymorphism with GC would further consolidate our previous study, which has demonstrated that DAL-1 gene is involved in anti-proliferation, inhibition of metastasis, associated with poor survival in GC [15–17].

Altogether, we have identified the association between the DAL-1 gene polymorphisms and GC susceptibility. The study has provided new evidence for DAL-1 gene as a potential target in GC clinical practice.

## Conclusion

This study demonstrated that the rs9953490 TA + AA genotypes of DAL-1 gene is significantly associated with the occurrence and development GC in the Han population of Northeast China.

## Supplementary Information

**Additional file 1**. **Supplementary Table 1:** Distribution of genotype and allele frequencies and their association with GC susceptibility. **Supplementary Table 2:** The frequencies of haplotypes of five SNPs in DAL-1 in cases and controls. **Supplementary Table 3:** Stratified analyses for the rs73937194 genotypes of DAL-1 gene in cases and controls. **Supplementary Table 4:** Stratified analyses for the rs3817466 genotypes of DAL-1 gene in cases and controls. **Supplementary Table 5:** Stratified analyses for the rs8082898 genotypes of DAL-1 gene in cases and controls. **Supplementary Table 6:** Stratified analyses for the rs73381527 genotypes of DAL-1 gene in cases and controls. **Supplementary Table 7:** Stratified analyses for the rs9953490 genotypes of DAL-1 gene in cases and controls. **Supplementary Table 8:** Association between DAL-1 (rs73937194) genotypes and clinicopathologic features of GC. **Supplementary Table 9:** Association between DAL-1 (rs3817466) genotypes and clinicopathologic features of GC. **Supplementary Table 10:** Association between DAL-1 (rs8082898) genotypes and clinicopathologic features of GC. **Supplementary Table 11:** Association between DAL-1 (rs73381527) genotypes and clinicopathologic characteristics features of GC. **Supplementary Table 12:** Association between DAL-1 (rs9953490) genotypes and clinicopathologic features of GC.

## Data Availability

All data generated or analyzed during this study are included in this published article and its Additional file 1.

## Abbreviations

GC: Gastric Cancer
DAL-1: Differentially expressed in adenocarcinoma of the lung-1
LOH: Loss of heterozygosity
EMT: Epithelial to mesenchymal transition
SNP: Single nucleotide polymorphism
MAF: Minor allele frequency
iMLDR: Improved Multiple Ligase Detection Reaction
PCRs: Polymerase chain reactions
ORs: Odds ratios
CIs: Confidence intervals
LD: Linkage disequilibrium
AJCC: American Joint Committee on Cancer
