# Supplementary material for: The rs9953490 polymorphism of DAL-1 gene is associated with gastric cancer risk in the Han population in Northeast China
Source: BMC Gastroenterol. 2021 Sep 27;21:354. doi: 10.1186/s12876-021-01929-9 (PMC8477503; doi:10.1186/s12876-021-01929-9)
Supplement: Supplementary file 1 — Additional file 1. Supplementary Table 1: Distribution of genotype and allele frequencies and their association with GC susceptibility. Supplementary Table 2: The frequencies of haplotypes of five SNPs in DAL-1 in cases and controls. Supplementary Table 3: Stratified analyses for the rs73937194 genotypes of DAL-1 gene in cases and controls. Supplementary Table 4: Stratified analyses for the rs3817466 genotypes of DAL-1 gene in cases and controls. Supplementary Table 5: Stratified analyses for the rs8082898 genotypes of DAL-1 gene in cases and controls. Supplementary Table 6: Stratified analyses for the rs73381527 genotypes of DAL-1 gene in cases and controls. Supplementary Table 7: Stratified analyses for the rs9953490 genotypes of DAL-1 gene in cases and controls. Supplementary Table 8: Association between DAL-1 (rs73937194) genotypes and clinicopathologic features of GC. Supplementary Table 9: Association between DAL-1 (rs3817466) genotypes and clinicopathologic features of GC. Supplementary Table 10: Association between DAL-1 (rs8082898) genotypes and clinicopathologic features of GC. Supplementary Table 11: Association between DAL-1 (rs73381527) genotypes and clinicopathologic characteristics features of GC. Supplementary Table 12: Association between DAL-1 (rs9953490) genotypes and clinicopathologic features of GC. [file 12876_2021_1929_MOESM1_ESM.docx]

The rs9953490 polymorphism of DAL-1 gene is associated with gastric cancer risk in the Han population in Northeast China

Hui Wang^1,2#^, Yuling Jiang^1,3#^, Lina Yu^1^, Lidan Xu^4^, Rongwei Guan^4^, Mengdi Cai^4^, Kexian Dong^4^, Xiao Liang^4^, Jing Bai^4^, Jingcui Yu^1,4*^

^1^Scientific Research Centre, the Second Affiliated Hospital of Harbin Medical University, Harbin 150081, China

^2^Department of Blood Transfusion, the Second Affiliated Hospital of Harbin Medical University, Harbin 150081, China

^3^The clinical laboratory, the First Affiliated Hospital of Harbin Medical University, Harbin 150001, China

^4^ Key laboratory of preservation of human genetic resources and disease control in China (Harbin Medical University), Ministry of Education, Harbin 150081, China.

^#^These authors contributed equally to this work.

*Corresponding Author: Jingcui Yu, M.D., Ph.D.

Email: [yujingcui@ems.hrbmu.edu.cn](#mailto:yujingcui@ems.hrbmu.edu.cn)

Supplementary Table 1. Distribution of genotype and allele frequencies and their association with GC susceptibility

| Genotypes | Cases (N=505)  n % | Controls (N=544)  n % | OR (95% CI) | *P* |
| --- | --- | --- | --- | --- |
| rs73937194 |  |  |  |  |
| CC | 447 88.5 | 490 90.1 | 1 |  |
| CG | 55 10.9 | 54 9.9 | 1.12 (0.75-1.66) | 0.586 |
| GG | 3 0.6 | 0 0 | - | 0.070 |
| Dominant |  |  |  |  |
| CC | 447 88.5 | 490 90.1 |  |  |
| CG+GG | 58 11.5 | 54 9.9 | 1.18 (0.80-1.74) | 0.414 |
| Recessive |  |  |  |  |
| CC+CG | 502 99.4 | 544 100 |  |  |
| GG | 3 0.6 | 0 0 | - | 0.072 |
| Allele |  |  |  |  |
| C | 949 94.0 | 1034 95.0 |  |  |
| G | 61 6.0 | 54 5.0 | 1.23 (0.84-1.79) | 0.279 |
| HWE | 0.364 | 0.223 |  |  |
| rs3817466 |  |  |  |  |
| GG | 407 80.6 | 446 82.0 | 1 |  |
| GA | 91 18.0 | 91 16.7 | 1.10 (0.80-1.51) | 0.575 |
| AA | 7 1.4 | 7 1.3 | 1.10 (0.38-3.15) | 0.865 |
| Dominant |  |  |  |  |
| GG | 407 80.6 | 446 82.0 |  |  |
| GA+AA | 98 19.4 | 98 18.0 | 1.10 (0.80-1.49) | 0.564 |
| Recessive |  |  |  |  |
| GG+GA | 498 98.6 | 537 98.7 |  |  |
| AA | 7 1.4 | 7 1.3 | 1.08 (0.38-3.10) | 0.889 |
| Allele |  |  |  |  |
| G | 905 89.6 | 983 90.3 |  |  |
| A | 105 10.4 | 105 9.7 | 1.09 (0.82-1.44) | 0.570 |
| HWE | 0.461 | 0.342 |  |  |
| rs8082898 |  |  |  |  |
| TT | 422 83.6 | 464 85.3 | 1 |  |
| TC | 79 15.6 | 76 14.0 | 1.14 (0.81-1.61) | 0.443 |
| CC | 4 0.8 | 4 0.7 | 1.10 (0.27-4.42) | 0.894 |
| Dominant |  |  |  |  |
| TT | 422 83.6 | 464 85.3 |  |  |
| TC+CC | 83 16.4 | 80 14.7 | 1.14 (0.82-1.59) | 0.440 |
| Recessive |  |  |  |  |
| TT+TC | 501 99.2 | 540 99.3 |  |  |
| CC | 4 0.8 | 4 0.7 | 1.08 (0.27-4.33) | 0.916 |
| Allele |  |  |  |  |
| T | 923 91.4 | 1004 92.3 |  |  |
| C | 87 8.6 | 84 7.7 | 1.13 (0.82-1.54) | 0.455 |
| HWE | 0.886 | 0.649 |  |  |
| rs73381527 |  |  |  |  |
| TT | 429 85.0 | 468 86.0 | 1 |  |
| TC | 75 14.9 | 76 14.0 | 1.08 (0.76-1.52) | 0.675 |
| CC | 1 0.1 | 0 0 | - | 0.297 |
| Dominant |  |  |  |  |
| TT | 429 85.0 | 468 86.0 |  |  |
| TC+CC | 76 15.0 | 76 14.0 | 1.09 (0.77-1.54) | 0.62 |
| Recessive |  |  |  |  |
| TT+TC | 504 99.8 | 544 100 |  |  |
| CC | 1 0.2 | 0 0 | - | 0.299 |
| Allele |  |  |  |  |
| T | 933 92.4 | 1012 93.0 |  |  |
| C | 77 7.6 | 76 7.0 | 1.10 (0.79-1.53) | 0.574 |
| HWE | 0.221 | 0.080 |  |  |
| rs9953490 |  |  |  |  |
| TT | 439 86.9 | 480 88.2 | 1 |  |
| TA | 64 12.7 | 60 11.0 | 1.17 (0.80-1.70) | 0.421 |
| AA | 2 0.4 | 4 0.8 | 0.55 (0.10-2.93) | 0.480 |
| Dominant |  |  |  |  |
| TT | 439 86.9 | 480 88.2 |  |  |
| TA+AA | 66 13.1 | 64 11.8 | 1.13 (0.78-1.63) | 0.522 |
| Recessive |  |  |  |  |
| TT+TA | 503 99.6 | 540 99.3 |  |  |
| AA | 2 0.4 | 4 0.7 | 0.54 (0.10-2.87) | 0.467 |
| Allele |  |  |  |  |
| T | 942 93.3 | 1020 93.8 |  |  |
| A | 68 6.7 | 68 6.2 | 1.08 (0.76-1.53) | 0.654 |
| HWE | 0.838 | 0.170 |  |  |

Notes: CI, confidence interval; OR, odd ratio；HWE: Hardy-Weinberg equilibrium

Chi square test for genotype and allele distributions between cases and controls

Supplementary Table 2. The frequencies of haplotypes of five SNPs in DAL-1 in cases and controls

| Haplotype | Frequency | Haplotype frequencies in GC | Haplotype frequencies in HC | Chi-Square | *P* |
| --- | --- | --- | --- | --- | --- |
| Block1 |  |  |  |  |  |
| GTTC | 0.777 | 0.765 | 0.789 | 1.688 | 0.194 |
| ACTC | 0.074 | 0.077 | 0.071 | 0.231 | 0.631 |
| GTCC | 0.068 | 0.071 | 0.066 | 0.242 | 0.623 |
| GTTG | 0.044 | 0.048 | 0.040 | 0.687 | 0.407 |
| ATTC | 0.020 | 0.020 | 0.020 | 0.004 | 0.947 |

Supplementary Table 3. Stratified analyses for the rs73937194 genotypes of DAL-1 gene in cases and controls

| Variables | rs73937194 | | | | | | | | | | | | |  |  |  |
| --- | --- | --- | --- | --- | --- | --- | --- | --- | --- | --- | --- | --- | --- | --- | --- | --- |
|  | Dominant | | | | | |  | Recessive | | | | | |  |  |  |
|  | CG+GG  (cases/controls) | | CC  (cases/controls) | | OR (95% CI) | *P* |  | GG  (cases/controls) | | CC+CG  (cases/controls) | | OR (95% CI) | *P* | | |  |
| Age |  |  |  |  |  |  |  |  |  |  |  |  |  | |  |  |
| ≤58 | 23 | 26 | 208 | 238 | 1.01 (0.56-1.83） | 0.968 |  | 0 | 0 | 231 | 264 | - | - | | | |
| >58 | 35 | 28 | 238 | 252 | 1.32 (0.78-2.24） | 0.297 |  | 3 | 0 | 270 | 280 | - | 0.079 | | | |
| Gender |  |  |  |  |  |  |  |  |  |  |  |  |  | | | |
| Male | 43 | 36 | 327 | 350 | 1.28 (0.8-2.04） | 0.302 |  | 2 | 0 | 368 | 386 | - | 0.148 | | | |
| Female | 15 | 18 | 120 | 140 | 0.97 (0.47-2.01） | 0.939 |  | 1 | 0 | 134 | 158 | - | 0.279 | | | |
| Smoking status |  |  |  |  |  |  |  |  |  |  |  |  |  | | | |
| Nonsmoker | 24 | 37 | 215 | 383 | 1.16 (0.67-1.98） | 0.600 |  | 2 | 0 | 237 | 420 | - | 0.060 | | | |
| Smoker | 34 | 17 | 232 | 107 | 0.92 (0.49-1.72） | 0.800 |  | 1 | 0 | 265 | 124 | - | 0.494 | | | |
| Pack-years |  |  |  |  |  |  |  |  |  |  |  |  |  | | | |
| 0 | 24 | 42 | 215 | 376 | 1 (0.59-1.7） | 0.998 |  | 2 | 0 | 237 | 418 | - | 0.061 | | | |
| ≤25 | 9 | 0 | 68 | 27 | - | 0.063 |  | 1 | 0 | 76 | 27 | - | 0.552 | | | |
| ＞25 | 25 | 12 | 164 | 87 | 1.11 (0.53-2.31） | 0.790 |  | 0 | 0 | 189 | 99 | - | - | | | |
| Drinking status |  |  |  |  |  |  |  |  |  |  |  |  |  | | | |
| Nondrinker | 32 | 39 | 273 | 376 | 1.13 (0.69-1.85） | 0.627 |  | 2 | 0 | 303 | 415 | - | 0.099 | | | |
| Drinker | 26 | 15 | 174 | 114 | 1.14 (0.58-2.24） | 0.713 |  | 1 | 0 | 199 | 129 | - | 0.421 | | | |

Notes: CI, confidence interval; OR, odd ratio

Supplementary Table 4. Stratified analyses for the rs3817466 genotypes of DAL-1 gene in cases and controls

| Variables | rs3817466 | | | | | | | | | | | | |  |  |  |
| --- | --- | --- | --- | --- | --- | --- | --- | --- | --- | --- | --- | --- | --- | --- | --- | --- |
|  | Dominant | | | | | |  | Recessive | | | | | |  |  |  |
|  | GA+AA  (cases/controls) | | GG  (cases/controls) | | OR (95% CI) | *P* |  | AA  (cases/controls) | | GG+GA  (cases/controls) | | OR (95% CI) | *P* | | |  |
| Age |  |  |  |  |  |  |  |  |  |  |  |  |  | |  |  |
| ≤58 | 46 | 48 | 185 | 216 | 1.12 (0.71-1.75) | 0.624 |  | 4 | 4 | 227 | 260 | 1.15 (0.28-4.63） | 0.849 | | | |
| >58 | 51 | 50 | 222 | 230 | 1.06 (0.69-1.63) | 0.802 |  | 3 | 3 | 270 | 277 | 1.03 (0.21-5.13） | 0.975 | | | |
| Gender |  |  |  |  |  |  |  |  |  |  |  |  |  | | | |
| Male | 77 | 69 | 293 | 317 | 1.21 (0.84-1.73) | 0.307 |  | 4 | 5 | 366 | 381 | 0.83 (0.22-3.12） | 0.786 | | | |
| Female | 21 | 29 | 114 | 129 | 0.82 (0.44-1.52) | 0.526 |  | 3 | 2 | 132 | 156 | 1.77 (0.30-10.52） | 0.529 | | | |
| Smoking status |  |  |  |  |  |  |  |  |  |  |  |  |  | | | |
| Nonsmoker | 46 | 79 | 193 | 341 | 1.03 (0.69-1.54) | 0.890 |  | 7 | 6 | 232 | 414 | 2.08 (0.71-6.13） | 0.183 | | | |
| Smoker | 52 | 19 | 214 | 105 | 1.34 (0.76-2.38) | 0.314 |  | 0 | 1 | 266 | 123 | - | 0.143 | | | |
| Pack-years |  |  |  |  |  |  |  |  |  |  |  |  |  | | | |
| 0 | 46 | 74 | 193 | 344 | 1.11 (0.74-1.67) | 0.622 |  | 7 | 5 | 232 | 413 | 2.49 (0.81-7.65） | 0.111 | | | |
| ≤25 | 13 | 6 | 64 | 21 | 0.71 (0.24-2.10) | 0.537 |  | 0 | 1 | 77 | 26 | - | 0.09 | | | |
| ＞25 | 39 | 18 | 150 | 81 | 1.17 (0.63-2.18) | 0.620 |  | 0 | 1 | 189 | 98 | - | 0.166 | | | |
| Drinking status |  |  |  |  |  |  |  |  |  |  |  |  |  | | | |
| Nondrinker | 62 | 75 | 243 | 340 | 1.16 (0.80-1.68) | 0.446 |  | 5 | 6 | 300 | 409 | 1.14 (0.34-3.75） | 0.834 | | | |
| Drinker | 36 | 23 | 164 | 106 | 1.01 (0.57-1.80) | 0.969 |  | 2 | 1 | 198 | 128 | 1.29 (0.12-14.31） | 0.834 | | | |

Notes: CI, confidence interval; OR, odd ratio

Supplementary Table 5. Stratified analyses for the rs8082898 genotypes of DAL-1 gene in cases and controls

| Variables | rs8082898 | | | | | | | | | | | | |  |  |  |
| --- | --- | --- | --- | --- | --- | --- | --- | --- | --- | --- | --- | --- | --- | --- | --- | --- |
|  | Dominant | | | | | |  | Recessive | | | | | |  |  |  |
|  | TC+CC  (cases/controls) | | TT  (cases/controls) | | OR (95% CI) | *P* |  | CC  (cases/controls) | | TT+TC  (cases/controls) | | OR (95% CI) | *P* | | |  |
| Age |  |  |  |  |  |  |  |  |  |  |  |  |  | |  |  |
| ≤58 | 41 | 41 | 190 | 223 | 1.17 (0.73-1.89) | 0.508 |  | 3 | 2 | 228 | 262 | 1.72 (0.29-10.19) | 0.548 | | | |
| >58 | 41 | 39 | 232 | 241 | 1.09 (0.68-1.75) | 0.716 |  | 1 | 2 | 272 | 278 | 0.51 (0.05-5.43) | 0.578 | | | |
| Gender |  |  |  |  |  |  |  |  |  |  |  |  |  | | | |
| Male | 65 | 60 | 305 | 326 | 1.16 (0.79-1.70) | 0.454 |  | 2 | 3 | 368 | 383 | 0.69 (0.12-4.14) | 0.688 | | | |
| Female | 18 | 20 | 117 | 138 | 1.06 (0.54-2.10) | 0.864 |  | 2 | 1 | 133 | 157 | 2.36 (0.23-24.54) | 0.472 | | | |
| Smoking status |  |  |  |  |  |  |  |  |  |  |  |  |  | | | |
| Nonsmoker | 40 | 60 | 199 | 360 | 1.21 (0.78-1.86) | 0.399 |  | 4 | 3 | 235 | 417 | 2.37 (0.55-10.2) | 0.248 | | | |
| Smoker | 43 | 20 | 223 | 104 | 1 (0.56-1.79) | 0.993 |  | 0 | 1 | 266 | 123 | - | 0.143 | | | |
| Pack-years |  |  |  |  |  |  |  |  |  |  |  |  |  | | | |
| 0 | 40 | 60 | 199 | 358 | 1.2 (0.78-1.85) | 0.413 |  | 4 | 4 | 235 | 414 | 1.76 (0.44-6.98) | 0.42 | | | |
| ≤25 | 11 | 4 | 66 | 23 | 0.96 (0.28-3.31) | 0.946 |  | 0 | 0 | 77 | 27 | - | - | | | |
| ＞25 | 32 | 16 | 157 | 83 | 1.06 (0.55-2.04) | 0.868 |  | 0 | 0 | 189 | 99 | - | - | | | |
| Drinking status |  |  |  |  |  |  |  |  |  |  |  |  |  | | | |
| Nondrinker | 52 | 61 | 253 | 354 | 1.19 (0.8-1.79) | 0.392 |  | 2 | 3 | 303 | 412 | 0.91 (0.15-5.45) | 0.915 | | | |
| Drinker | 31 | 19 | 169 | 110 | 1.06 (0.57-1.97) | 0.849 |  | 2 | 1 | 198 | 128 | 1.29 (0.12-14.31) | 0.834 | | | |

Notes: CI, confidence interval; OR, odd ratio

Supplementary Table 6. Stratified analyses for the rs73381527 genotypes of DAL-1 gene in cases and controls

| Variables | rs73381527 | | | | | | | | | | | | |  |  |  |
| --- | --- | --- | --- | --- | --- | --- | --- | --- | --- | --- | --- | --- | --- | --- | --- | --- |
|  | Dominant | | | | | |  | Recessive | | | | | |  |  |  |
|  | TC+CC  (cases/controls) | | TT  (cases/controls) | | OR (95% CI) | *P* |  | CC  (cases/controls) | | TT+TC  (cases/controls) | | OR (95% CI) | *P* | | |  |
| Age |  |  |  |  |  |  |  |  |  |  |  |  |  | |  |  |
| ≤58 | 34 | 33 | 197 | 231 | 1.21 (0.72-2.02) | 0.472 |  | 0 | 0 | 231 | 264 | - | - | | | |
| >58 | 42 | 43 | 231 | 237 | 1 (0.63-1.59) | 0.993 |  | 1 | 0 | 272 | 280 | - | 0.311 | | | |
| Gender |  |  |  |  |  |  |  |  |  |  |  |  |  | | | |
| Male | 57 | 49 | 313 | 337 | 1.25 (0.83-1.89) | 0.283 |  | 1 | 0 | 369 | 386 | - | 0.307 | | | |
| Female | 19 | 27 | 116 | 131 | 0.79 (0.42-1.5) | 0.480 |  | 0 | 0 | 135 | 158 | - | - | | | |
| Smoking status |  |  |  |  |  |  |  |  |  |  |  |  |  | | | |
| Nonsmoker | 37 | 63 | 202 | 357 | 1.04 (0.67-1.61) | 0.869 |  | 0 | 0 | 239 | 420 | - | - | | | |
| Smoker | 39 | 13 | 227 | 111 | 1.47 (0.75-2.85) | 0.258 |  | 1 | 0 | 265 | 124 | - | 0.494 | | | |
| Pack-years |  |  |  |  |  |  |  |  |  |  |  |  |  | | | |
| 0 | 37 | 62 | 202 | 356 | 1.05 (0.68-1.64) | 0.823 |  | 0 | 0 | 239 | 418 | - | - | | | |
| ≤25 | 10 | 6 | 67 | 21 | 0.52 (0.17-1.59) | 0.252 |  | 0 | 0 | 77 | 27 | - | - | | | |
| ＞25 | 29 | 8 | 160 | 91 | 2.06 (0.92-4.64) | 0.080 |  | 1 | 0 | 188 | 99 | - | 0.468 | | | |
| Drinking status |  |  |  |  |  |  |  |  |  |  |  |  |  | | | |
| Nondrinker | 44 | 61 | 261 | 354 | 0.98 (0.64-1.49) | 0.918 |  | 0 | 0 | 305 | 415 | - | - | | | |
| Drinker | 32 | 15 | 168 | 114 | 1.45 (0.75-2.79) | 0.269 |  | 1 | 0 | 199 | 129 | - | 0.421 | | | |

Notes: CI, confidence interval; OR, odd ratio

| Variables |  |  |  |  |  |  |  |  |  |
| --- | --- | --- | --- | --- | --- | --- | --- | --- | --- |
|  | Recessive | | | | | |  |  |  |
|  | CC  (cases/controls) | | TT+TC  (cases/controls) | | OR (95% CI) | *P* | | |  |
| Age |  |  |  |  |  |  | |  |  |
| ≤58 | 1 | 2 | 230 | 262 | 0.57 (0.05-6.13) | 0.642 | | | |
| >58 | 1 | 2 | 272 | 278 | 0.51 (0.05-5.43) | 0.578 | | | |
| Gender |  |  |  |  |  |  | | | |
| Male | 2 | 4 | 368 | 382 | 0.52 (0.1-2.77) | 0.443 | | | |
| Female | 0 | 0 | 135 | 158 | - | - | | | |
| Smoking status |  |  |  |  |  |  | | | |
| Nonsmoker | 2 | 3 | 237 | 417 | 1.17 (0.19-7.06) | 0.862 | | | |
| Smoker | 0 | 1 | 266 | 123 | - | 0.143 | | | |
| Pack-years |  |  |  |  |  |  | | | |
| 0 | 2 | 3 | 237 | 415 | 1.17 (0.19-7.02) | 0.866 | | | |
| ≤25 | 0 | 0 | 77 | 27 | - | - | | | |
| ＞25 | 0 | 1 | 189 | 98 | - | 0.166 | | | |
| Drinking status |  |  |  |  |  |  | | | |
| Nondrinker | 2 | 3 | 303 | 412 | 0.91 (0.15-5.45) | 0.915 | | | |
| Drinker | 0 | 1 | 200 | 128 | - | 0.212 | | | |

Supplementary Table 7. Stratified analyses for the rs9953490 genotypes of DAL-1 gene in cases and controls

Notes: CI, confidence interval; OR, odd ratio

Supplementary Table 8. Association between DAL-1 (rs73937194) genotypes and clinicopathologic features of GC

| Variables | rs73937194 | | | | | | | | |  |  |  |
| --- | --- | --- | --- | --- | --- | --- | --- | --- | --- | --- | --- | --- |
|  | Dominant | | | |  | Recessive | | | |  |  |  |
|  | CG+GG | CC | OR (95% CI) | *P* |  | CG+CC | GG | OR (95% CI) | *P* | | |  |
| Tumor size (cm) |  |  |  |  |  |  |  |  |  | |  |  |
| <5 | 19 | 142 |  |  |  | 160 | 1 |  |  | | | |
| ≥ 5 | 13 | 100 | 0.97 (0.46-2.06) | 0.940 |  | 113 | 0 | - | 0.401 | | | |
| Neoplasia location |  |  |  |  |  |  |  |  |  | | | |
| Non-cardia | 28 | 210 |  |  |  | 237 | 1 |  |  | | | |
| Cardia | 4 | 32 | 0.94 (0.31-2.85) | 0.909 |  | 36 | 0 | - | 0.697 | | | |
| Invasion depth |  |  |  |  |  |  |  |  |  | | | |
| T1-T2 | 8 | 82 |  |  |  | 89 | 1 |  |  | | | |
| T3-T4 | 24 | 160 | 1.54 (0.66-3.56) | 0.315 |  | 184 | 0 | - | 0.152 | | | |
| Lymph metastasis |  |  |  |  |  |  |  |  |  | | | |
| N0 | 17 | 96 |  |  |  | 112 | 1 |  |  | | | |
| N1 | 6 | 66 | 0.51 (0.19-1.35) | 0.177 |  | 72 | 0 | - | 0.423 | | | |
| N2 | 6 | 58 | 0.58 (0.22-1.55) | 0.281 |  | 64 | 0 | - | 0.450 | | | |
| N3 | 3 | 22 | 0.77 (0.21-2.85) | 0.696 |  | 25 | 0 | - | 0.637 | | | |
| TNM stage |  |  |  |  |  |  |  |  |  | | | |
| I-Ⅱ | 22 | 168 |  |  |  | 189 | 1 |  |  | | | |
| Ⅲ | 10 | 74 | 1.03 (0.47-2.29) | 0.938 |  | 84 | 0 | - | 0.505 | | | |
| Lauren’s classification |  |  |  |  |  |  |  |  |  | | | |
| Intestinal | 27 | 176 |  |  |  | 202 | 1 |  |  | | | |
| Diffuse | 5 | 66 | 0.49 (0.19-1.31) | 0.158 |  | 71 | 0 | - | 0.554 | | | |

Notes: CI, confidence interval; OR, odd ratio

Supplementary Table 9. Association between DAL-1 (rs3817466) genotypes and clinicopathologic features of GC

| Variables | rs3817466 | | | | | | | | |  |  |  |
| --- | --- | --- | --- | --- | --- | --- | --- | --- | --- | --- | --- | --- |
|  | Dominant | | | |  | Recessive | | | |  |  |  |
|  | GA+AA | GG | OR (95% CI) | *P* |  | GA+GG | AA | OR (95% CI) | *P* | | |  |
| Tumor size (cm) |  |  |  |  |  |  |  |  |  | |  |  |
| <5 | 32 | 129 |  |  |  | 161 | 0 |  |  | | | |
| ≥ 5 | 26 | 87 | 1.20 (0.67-2.16) | 0.532 |  | 110 | 3 | - | 0.038 | | | |
| Neoplasia location |  |  |  |  |  |  |  |  |  | | | |
| Non-cardia | 50 | 188 |  |  |  | 235 | 3 |  |  | | | |
| Cardia | 8 | 28 | 1.07 (0.46-2.50) | 0.868 |  | 36 | 0 | - | 0.498 | | | |
| Invasion depth |  |  |  |  |  |  |  |  |  | | | |
| T1-T2 | 18 | 72 |  |  |  | 89 | 1 |  |  | | | |
| T3-T4 | 40 | 144 | 1.11 (0.60-2.07) | 0.741 |  | 182 | 2 | 1.02 (0.09-11.43) | 0.986 | | | |
| Lymph metastasis |  |  |  |  |  |  |  |  |  | | | |
| N0 | 23 | 90 |  |  |  | 113 | 0 |  |  | | | |
| N1 | 15 | 57 | 1.03 (0.50-2.14) | 0.937 |  | 70 | 2 | - | 0.075 | | | |
| N2 | 14 | 50 | 1.10 (0.52-2.32) | 0.811 |  | 63 | 1 | - | 0.183 | | | |
| N3 | 6 | 19 | 1.24 (0.44-3.44) | 0.686 |  | 25 | 0 | - | - | | | |
| TNM stage |  |  |  |  |  |  |  |  |  | | | |
| I-Ⅱ | 37 | 153 |  |  |  | 188 | 2 |  |  | | | |
| Ⅲ | 21 | 63 | 1.38 (0.75-2.53) | 0.302 |  | 83 | 1 | 0.88 (0.08-9.86) | 0.919 | | | |
| Lauren’s classification |  |  |  |  |  |  |  |  |  | | | |
| Intestinal | 44 | 159 |  |  |  | 200 | 3 |  |  | | | |
| Diffuse | 14 | 57 | 0.89 (0.45-1.74) | 0.728 |  | 71 | 0 | - | 0.303 | | | |

Notes: CI, confidence interval; OR, odd ratio

Supplementary Table 10. Association between DAL-1 (rs8082898) genotypes and clinicopathologic features of GC

| Variables | rs8082898 | | | | | | | | |  |  |  |
| --- | --- | --- | --- | --- | --- | --- | --- | --- | --- | --- | --- | --- |
|  | Dominant | | | |  | Recessive | | | |  |  |  |
|  | TC+CC | TT | OR (95% CI) | *P* |  | TC+TT | CC | OR (95% CI) | *P* | | |  |
| Tumor size (cm) |  |  |  |  |  |  |  |  |  | |  |  |
| <5 | 24 | 137 |  |  |  | 161 | 0 |  |  | | | |
| ≥ 5 | 22 | 91 | 1.38 (0.73-2.60) | 0.320 |  | 111 | 2 | - | 0.090 | | | |
| Neoplasia location |  |  |  |  |  |  |  |  |  | | | |
| Non-cardia | 39 | 199 |  |  |  | 236 | 2 |  |  | | | |
| Cardia | 7 | 29 | 1.23 (0.50-3.01) | 0.647 |  | 36 | 0 | - | 0.581 | | | |
| Invasion depth |  |  |  |  |  |  |  |  |  | | | |
| T1-T2 | 14 | 76 |  |  |  | 90 | 0 |  |  | | | |
| T3-T4 | 32 | 152 | 1.14 (0.58-2.27) | 0.703 |  | 182 | 2 | - | 0.321 | | | |
| Lymph metastasis |  |  |  |  |  |  |  |  |  | | | |
| N0 | 18 | 95 |  |  |  | 113 | 0 |  |  | | | |
| N1 | 13 | 59 | 1.16 (0.53-2.55) | 0.706 |  | 70 | 2 | - | 0.075 | | | |
| N2 | 11 | 53 | 1.10 (0.48-2.49) | 0.828 |  | 64 | 0 | - | - | | | |
| N3 | 4 | 21 | 1.01 (0.31-3.28) | 0.993 |  | 25 | 0 | - | - | | | |
| TNM stage |  |  |  |  |  |  |  |  |  | | | |
| I-Ⅱ | 30 | 160 |  |  |  | 189 | 1 |  |  | | | |
| Ⅲ | 16 | 68 | 1.25 (0.64-2.45) | 0.506 |  | 83 | 1 | 0.44 (0.03-6.59) | 0.552 | | | |
| Lauren’s classification |  |  |  |  |  |  |  |  |  | | | |
| Intestinal | 34 | 169 |  |  |  | 201 | 2 |  |  | | | |
| Diffuse | 12 | 59 | 1.01 (0.49-2.08) | 0.976 |  | 71 | 0 | - | 0.401 | | | |

Notes: CI, confidence interval; OR, odd ratio

Supplementary Table 11. Association between DAL-1 (rs73381527) genotypes and clinicopathologic characteristicsfeatures of GC

| Variables | rs73381527 | | | | | | | | |  |  |  |
| --- | --- | --- | --- | --- | --- | --- | --- | --- | --- | --- | --- | --- |
|  | Dominant | | | |  | Recessive | | | |  |  |  |
|  | TC+CC | TT | OR (95% CI) | *P* |  | TC+TT | CC | OR (95% CI) | *P* | | |  |
| Tumor size (cm) |  |  |  |  |  |  |  |  |  | |  |  |
| <5 | 20 | 141 |  |  |  | 160 | 1 |  |  | | | |
| ≥ 5 | 18 | 95 | 1.34 (0.67-2.65) | 0.408 |  | 113 | 0 | - | 0.401 | | | |
| Neoplasia location |  |  |  |  |  |  |  |  |  | | | |
| Non-cardia | 30 | 208 |  |  |  | 237 | 1 |  |  | | | |
| Cardia | 8 | 28 | 1.98 (0.84-4.69) | 0.120 |  | 36 | 0 | - | 0.697 | | | |
| Invasion depth |  |  |  |  |  |  |  |  |  | | | |
| T1-T2 | 10 | 80 |  |  |  | 90 | 0 |  |  | | | |
| T3-T4 | 28 | 156 | 1.44 (0.67-3.09) | 0.356 |  | 183 | 1 | - | 0.484 | | | |
| Lymph metastasis |  |  |  |  |  |  |  |  |  | | | |
| N0 | 14 | 99 |  |  |  | 113 | 0 |  |  | | | |
| N1 | 10 | 62 | 1.14 (0.48-2.73) | 0.767 |  | 71 | 1 | - | 0.209 | | | |
| N2 | 10 | 54 | 1.31 (0.55-3.14) | 0.546 |  | 64 | 0 | - | - | | | |
| N3 | 4 | 21 | 1.35 (0.40-4.49) | 0.628 |  | 25 | 0 | - | - | | | |
| TNM stage |  |  |  |  |  |  |  |  |  | | | |
| I-Ⅱ | 26 | 164 |  |  |  | 189 | 1 |  |  | | | |
| Ⅲ | 12 | 72 | 1.05 (0.50-2.20) | 0.894 |  | 84 | 0 | - | 0.505 | | | |
| Lauren’s classification |  |  |  |  |  |  |  |  |  | | | |
| Intestinal | 25 | 178 |  |  |  | 202 | 1 |  |  | | | |
| Diffuse | 13 | 58 | 1.60 (0.77-3.31) | 0.208 |  | 71 | 0 | - | 0.554 | | | |

Notes: CI, confidence interval; OR, odd ratio

| Variables | rs9953490 | | | |
| --- | --- | --- | --- | --- |
|  | Recessive | | | |
|  | TA+TT | AA | OR (95% CI) | *P* |
| Tumor size (cm) |  |  |  |  |
| <5 | 161 | 0 |  |  |
| ≥ 5 | 112 | 1 | - | 0.232 |
| Neoplasia location |  |  |  |  |
| Non-cardia | 237 | 1 |  |  |
| Cardia | 36 | 0 | - | 0.697 |
| Invasion depth |  |  |  |  |
| T1-T2 | 89 | 1 |  |  |
| T3-T4 | 184 | 0 | - | 0.152 |
| Lymph metastasis |  |  |  |  |
| N0 | 113 | 0 |  |  |
| N1 | 72 | 0 | - | - |
| N2 | 63 | 1 | - | 0.183 |
| N3 | 25 | 0 | - | - |
| TNM stage |  |  |  |  |
| I-Ⅱ | 189 | 1 |  |  |
| Ⅲ | 84 | 0 | - | 0.505 |
| Lauren’s classification |  |  |  |  |
| Intestinal | 202 | 1 |  |  |
| Diffuse | 71 | 0 | - | 0.554 |

Supplementary Table 12. Association between DAL-1 (rs9953490) genotypes and clinicopathologic features of GC

Notes: CI, confidence interval; OR, odd ratio
